# Supplementary material for: Cerebroventricular Injection of Pgk1 Attenuates MPTP-Induced Neuronal Toxicity in Dopaminergic Cells in Zebrafish Brain in a Glycolysis-Independent Manner
Source: Int J Mol Sci. 2022 Apr 8;23(8):4150. doi: 10.3390/ijms23084150 (PMC9025024; doi:10.3390/ijms23084150)
Supplement: Supplementary file 1 [file ijms-23-04150-s001.zip › ijms-1602654-supplementary.pdf]

Supplemental Data

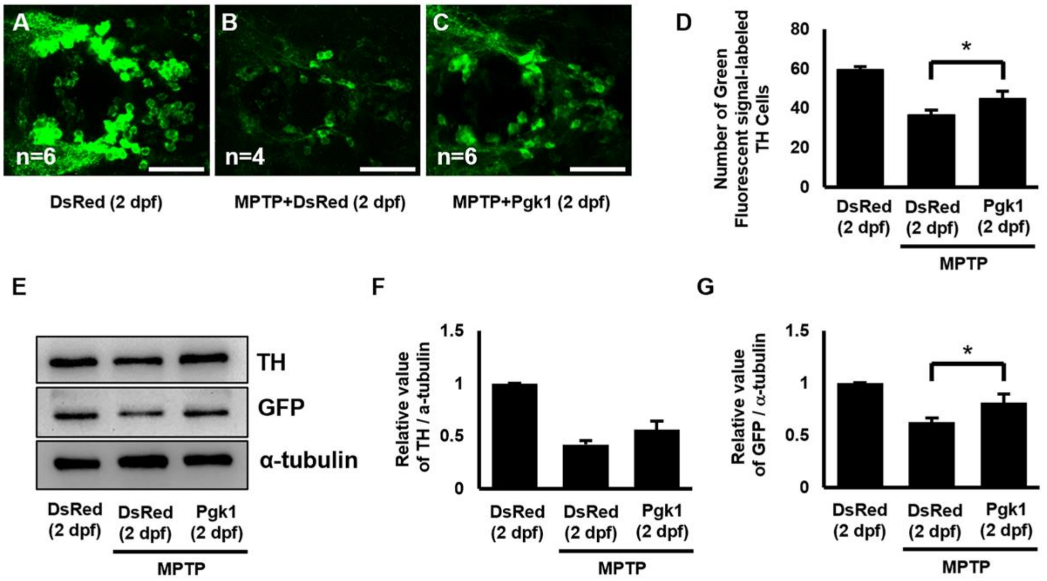

Figure S1. Direct injection of Pgk1 into brain prevents MPTP-induced dopaminergic cell death in the ventral diencephalon of zebrafish embryo at 4 dpf. (**A-C**) Using confocal microscopy, Tyrosine hydroxylase (TH)-specific antibody labeled with green fluorescence signal could be observed in the vDC region of embryos with different treatments as indicated. (**A**) DsRed (2 dpf): embryos at 2 dpf were injected with DsRed protein without MPTP treatment as a negative control; (**B**) MPTP+DsRed (2 dpf): embryos at 2 dpf were injected with DsRed protein and treated with 45 M MPTP from 2 through 4 dpf as a mock control; (**C**) MPTP+Pgk1 (2 dpf): embryos at 2 dpf were injected with Pgk1 and treated with 45 μM MPTP from 2 through 4 dpf as an experimental group. Number of examined embryos in each group was indicated at the lower left corner of each panel. Projections of Z-stack images were generated with 2 μm. Scale bar: 50 μm. (**D**) Statistical analysis of the average number of green fluorescent signal-labeled TH cells in vDC obtained from three groups. (**E**) Western blot analysis of the TH and GFP proteins expressed in the head of larvae from transgenic line Tg(*dat:EGFP*) treated as indicated. The α-tubulin served as an internal control. The relative expression values of (**F**) TH and (**G**) GFP quantified from different

groups after normalization of the expression level of α-tubulin. The level of each examined protein expressed in the negative group set as 1. All data were averaged from three independent experiments and represented as mean±S.D. Student's *t*-test was used to determine significant differences between each group (*, p<0.05).

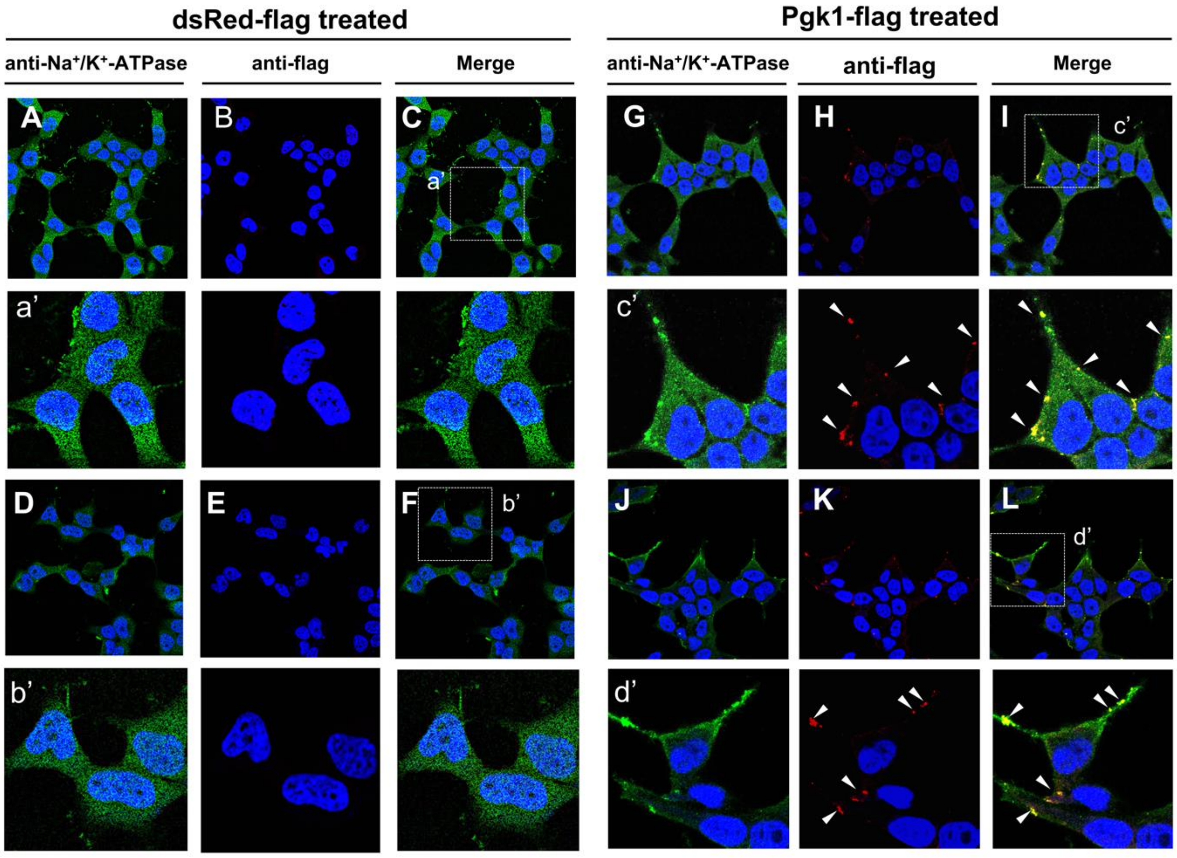

Figure S2. Extracellular addition of Pgk1 was located at the cell membrane of neural cells. The green fluorescent signal was used to label Na^+/K^+-ATPase (membrane marker; positive control), while the red fluorescent signal was used to label DsRed-Flag (negative control). (A-F) The culture medium of NSC34 was added with 33 ng/μl DsRed-Flag. No red fluorescent signal located at the neural cell membrane was observed. (G-L) The culture medium of NSC34 was added with 33 ng/μl Pgk1-Flag. The red fluorescent signal located at neural cell membrane was colocalized with green fluorescent-labeled Na^+/K^+-ATPase (indicated by white

arrowheads), suggesting that ePgk1 may be associated with a membrane protein of NSC34 neural cells. Panels a'-d' are amplified from the area indicated by a box on the panels C, F, I and L, respectively.

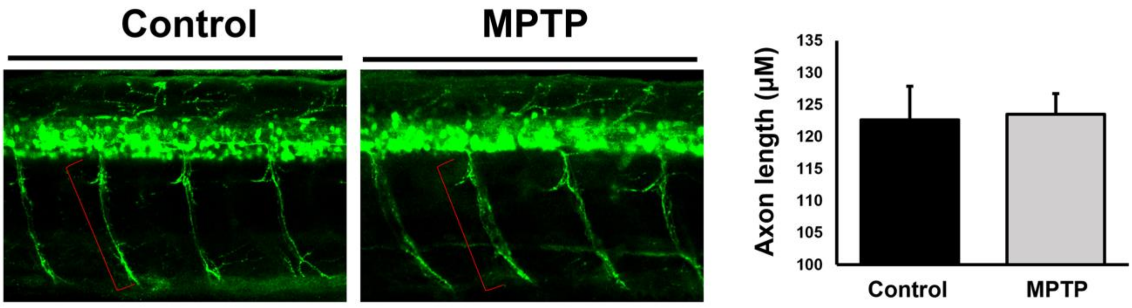

Figure S3. The effect of MPTP treatment on the development of zebrafish motor neurons. (**A**) The embryos from transgenic line *Tg(mnx:GFP),* in which the motor neurons were specifically tagged with GFP, were treated with 45 μM MPTP from 2 through 6 dpf. The GFP-tagged motor neuron of embryos was observed under fluorescent microscopy (indicated by bracelets). The phenotype of caudal primary motor neurons of 6-dpf embryos from the untreated control and MPTP-treated embryos was studied. (**B**) The neurite length (in μm) was measured and compared.

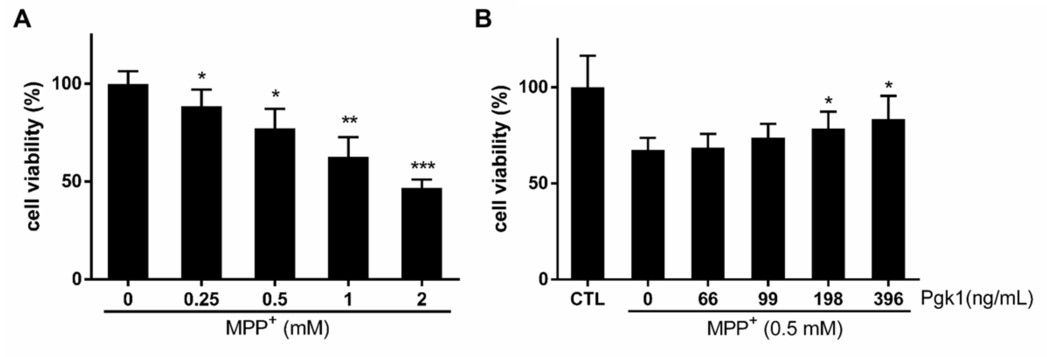

Figure S4. Extracellular addition of Pgk1 prevents MPP^{+}-induced SH-SY5Y cell death. (**A**) Survival rate of SH-SY5Y cells incubated with different

concentrations, ranging from 0.25 to 2 mM, as indicated, of MPP^+ for 24 h. Cell viability was calculated by hemocytometer assay. The cell survival rate of each group was determined in comparison to the untreated control baseline, which was set as 100%. (**B**) The survival rate of SH-SY5Y cells incubated with 0.5 mM MPP^+ combined with different concentrations of Pgk1 as indicated for 24 h. The cells treated with DMSO served as control group (CTL) set as 100% of cell viability. The cell survival rate of each group was determined in comparison to control group baseline. Each group (well) was counted three times. All data were averaged from three independent experiments and represented as mean±S.D. Student's *t*-test was used to determine significant differences between each group (*, $p<0.05$; **, $p<0.01$; ***, $p<0.001$).
